# Editorial: Cell Signaling in Tumors of Endocrine and Endocrine-Responsive Tissues

**DOI:** 10.3389/fendo.2019.00211

**Published:** 2019-04-05

**Authors:** Claire Perks, Angela Hague

**Affiliations:** ^1^Insulin-like Growth Factors and Metabolic Endocrinology Group, Bristol Medical School, Southmead Hospital, Bristol, United Kingdom; ^2^Bristol Dental School, Bristol, United Kingdom

**Keywords:** cell signaling, endocrine tumor, targeted therapies, endocrine-responsive tumor, biomarkers

Cancers of endocrine tissues are relatively rare compared to many other solid tumors: they are diverse and include both genetically inherited and non-inherited forms, such as gastroenteropancreatic neuroendocrine tumors, that are clinically and biologically heterogeneous and as a result are difficult to treat. The article entitled “*Clinical and preclinical advances in gastroenteropancreatic neuroendocrine tumor therapy*” by Crabtree outlines current ongoing clinical trials testing emerging therapies for the disease, including immunotherapy, and antiangiogenic therapies. The review also discusses potential pre-clinical pathways, such as PI3K/Akt and genetic profiling that may lead to additional targeted approaches.

An understanding of the defects in signaling pathways in specific tumor types offers opportunities for targeted therapies, but also significant challenges due to their complexity. A review article by Xu et al. focuses on “*Sonic hedgehog (Shh) signaling in thyroid cancer.”* This review highlights the complexity of the Shh pathway by delineating the intricate cross-talk that occurs between Shh and the PI3K and MAPK pathways. The authors then focus on the key role of Shh signaling in the regulation of thyroid cancer stem cell (CSC) self-renewal. They conclude that a better understanding of the exact way in which Shh signaling is involved in CSC renewal and how it interacts with the PI3K/MAPK pathways requires more detailed investigation in order to develop novel therapies.

Stem cells are also discussed in relation to pituitary tumors in a review by Vankelecom and Roose entitled “*The stem cell connection of pituitary tumors*.” The authors provide and discuss evidence to suggest that “tumor stem cells” exist in pituitary tumors and the relationship between them and the role they play in the process of pituitary tumorigenesis, such as development and growth. The authors then describe the limited knowledge in pituitary tumors surrounding dysregulation of the main stem cell signaling pathways: for example NOTCH, Shh, and WNT signaling. The authors summarize that having a better understanding of the role that stem cells play in pituitary tumorigenesis could lead to novel drug targets and improved options for therapy.

The insulin-like growth factor axis is often dysregulated in tumorigenesis and is known to play a key role in the risk and progression of many cancers. It is also well-established that perturbations in the IGF axis are exacerbated in the presence of metabolic disturbance, such as obesity and type 2 diabetes. A review by Vella et al. entitled “*PPAR-gamma agonists as antineoplastic agents in cancers with dysregulated IGF axis”* succinctly describes the mechanisms underlying the link between obesity, insulin resistance, and cancer and discusses how PPAR-gamma agonists may act as anti-tumor drugs by inhibiting the very pathways that are activated and the phenotypes induced by components of the IGF axis. The authors discuss that whilst clinical trials for PPAR-gamma agonists have produced conflicting data, it might be that using better biomarkers for patient selection, such as disturbed metabolic status indicated by overactivation of the IGF axis, may provide a more targeted approach.

Whilst localized disease is clinically manageable, death from endocrine-responsive breast cancer is generally due to the spread of disease to sites other than the breast: As many as 30% of patients with early breast cancer develop this type of disease. Androgens have been shown to have differential effects on breast cancer cell phenotypes and an original article by Montt-Guevara et al. entitled “Androgens regulate T47D cells motility and invasion through actin cytoskeleton remodeling” describes a pro-tumorigenic role for androgens in promoting invasion and migration. They determined that the ability of androgens to achieve this was via altering actin organization that is mediated by Moesin. Identifying this role for androgens in promoting a metastatic phenotype, together with an underlying mechanism, may lead to novel approaches for treatment of breast cancer.

In conclusion, the “Cell Signaling in Tumors of Endocrine and Endocrine-responsive Tissues” research topic highlights the importance of understanding complex signaling pathways with a view to applying a more targeted approach to treatment.

## Author Contributions

All authors listed have made a substantial, direct and intellectual contribution to the work, and approved it for publication.

### Conflict of Interest Statement

The authors declare that the research was conducted in the absence of any commercial or financial relationships that could be construed as a potential conflict of interest.

